# Complete Genome Sequence and Characterization of a Polyethylene Biodegradation Strain, *Streptomyces Albogriseolus* LBX-2

**DOI:** 10.3390/microorganisms7100379

**Published:** 2019-09-22

**Authors:** Huanhuan Shao, Meiju Chen, Xueting Fei, Ronglin Zhang, Yue Zhong, Weimin Ni, Xiang Tao, XinYi He, Erliang Zhang, Bin Yong, Xuemei Tan

**Affiliations:** 1 College of Life Sciences, Sichuan Normal University, Chengdu 610101, China; wsshforget@163.com (H.S.); hexinyi64704748@163.com (X.H.);; 2 College of Life Sciences, Sichuan University, Chengdu 610064, China

**Keywords:** *Streptomyces albogriseolus* LBX-2, polyethylene biodegradation, genome, synteny comparison

## Abstract

A bacterial strain, *Streptomyces albogriseolus* LBX-2, was isolated from a soil sample in Chengdu, China. *S. albogriseolus* LBX-2 is an aerobic and Gram-positive microorganism that is capable of using the polyethylene as the sole carbon source. Results of scanning electron microscopy and tensile tests indicated that *S. albogriseolus* LBX-2 could cause the damages to polyethylene (PE). Suspension culture of LBX-2 resulted in the weight loss in the PE powder over a 15-day period. The bacterial growth curve assay clearly demonstrated the utilization of n-hexadecane and n-octadecane for the strain LBX-2. Phylogenetic analysis showed that it was grouped in the same clade as *S. albogriseolus* belonging to *Streptomyces*. The complete genome of strain LBX-2 consists of a chromosome of 7,210,477 bp and a linear plasmid of 336,677 bp. Compared with other strains of *Streptomyces*, the genome size of *S. albogriseolus* LBX-2 was smaller than the average but its guanine and cytosine content (72.47%) was higher than the others. The Non-Redundant Protein Database (NR), Kyoto Encyclopedia of Genes and Genomes (KEGG), SwissProt, Gene Ontology (GO) and Clusters of Orthologous Groups (COG) annotations provided information on the specific functions of encoded proteins. A total of 21 monooxygenase and 22 dioxygenase genes were found in its genome. Synteny comparison with the genome of *Streptomyces coelicolor* A3(2) revealed a low overall genetic diversity between them. This study provides valuable information to reveal the underlying mechanisms on PE degradation by *S. albogriseolus* LBX-2.

## 1. Introduction

Polyethylene (PE) is the most commonly used synthetic polymer known as plastics due to its high stability and inert compounds. However, its huge production and improper waste management have become a serious threat to terrestrial and marine environments [1,2]. In addition to the problems associated with the debris (i.e., ingestion or choking by marine animals), plastic products can also be broken into either micro- (diameter < 5 mm) or nano-plastic sizes (diameter < 1 μm) by chemical (e.g., hydrolysis) and physical (e.g., ultraviolet) aging under natural conditions [3]. Such micro- and nano-plastics are accumulated and dispersed in many environments. It was reported that micro-plastics have been numerically abundant in both aquatic and terrestrial environments [4,5,6,7]. They have affected the reproduction of oyster or caused the reduction of nutrient assimilation for marine organisms [8,9]. Moreover, microplastics can also adsorb hydrophobic compounds such as persistent organic pollutants (POPs) and contaminants, which could be accumulated in the larger consumers, endangering then the survival of many organisms through trophic transfers in the food web [10,11]. Further, ingestion of microplastics could also affect digestion by blocking of the digestive track and/or irritation of the epithelial lining [8,12].

To combat these growing problems associated with plastics pollution, three main solutions have been employed to facilitate degradation of PE, namely, photodegradation, thermo-oxidative, and biodegradation [13]. However, compared with the degradation photo-physical methods, biodegradation has been shown to be more efficient, cost-effective and environment-friendly. Microorganisms, especially bacteria and fungi, enable the depolymerization and remineralization of PE by secreting extracellular enzyme(s) as well as by the biofilms encased within an extracellular matrix composed of free nucleic acids, proteins, and polysaccharides [1,14].

Previous studies reported many potential PE degrading bacteria. For example, Sivan identified that *Brevibacillus* spp. and *Bacillus* spp. were capable of degrading PE [2]. Sangeetha Devi reported *Bacillus* spp. and *Pseudomonas* spp. capable of using PE as their major carbon and energy source for growth [15]. Most of the PE-degrading strains belong to *Pseudomonas*, *Streptomyces*, *Corynebacterium*, *Arthrobacter*, *Micrococcus*, and *Rhodococcus* [15,16]. Among the promising microbial strains for PE biodegradation, the actinomycete *Streptomyces* bacteria have a strong ability to decompose complex organic substances such as keratin, organic insecticides, cellulose, paraffin, and even PE [17,18,19,20]. In this paper, the complete genome of a PE-degrading strain *S. albogriseolus* LBX-2 was analyzed. Its PE degrading capability was also characterized. The results of this study are propitious to understand the mechanism of PE biodegradation and exploit its PE-degrading enzymes in the future.

## 2. Materials and Methods

### 2.1. Bacterial Strains and Culture Conditions

The soil filled with PE was collected from Sichuan Normal University in Chengdu, China. The samples were deposited in the sterile distilled water for making a series of dilutions. From each dilution, 100 μL was spread in the 100 mL medium A (tryptone 10 g, mineral oil 0.1 mL, dipotassium hydrogenphosphate 0.7 g, magnesium sulfate heptahydrate 0.7 g, ammonium nitrate 1 g, Sodium chloride 0.005 g, ferrous sulfate 0.002 g, zinc sulfate heptahydrate 0.002 g, manganese sulphate 0.001 g). Then, the culture liquid was spread on the Gause’s medium (potassium nitrate 1 g, potassium hydrogen phosphate 0.5 g, magnesium sulphate 0.5 g, ferrous sulphate 0.01 g, Sodium chloride 0.5 g), in which PE is the sole carbon source. After 16S rRNA and physiological and biochemical identification, one isolated bacterium was named *S. albogriseolus* LBX-2 [21]. A colony of *S. albogriseolus* LBX-2 was then picked and re-grown in a fresh 100 mL Gause’s medium containing only polyethylene sheet (10 cm × 3 cm) as the sole carbon source, and further incubated in flasks (250 mL) on a rotary shaker (180 rpm) at 30 °C for 45 days.

### 2.2. Scanning Electron Microscopy (SEM) and Tensile Assays

Pieces of the PE sheets were collected after 0, 15, 30, and 45 days of incubation with *S. albogriseolus* LBX-2 were taken out and washed with 0.01 M phosphate buffer (pH = 7.2) for 2 min to remove the residual media. The membranes were then immersed in 2% SDS solution overnight to remove the residual cells, and ultrasonically treated for 30 min. After which, the sheets were cleaned with distilled water at 40 °C 3 times to get rid of the SDS. Finally, 2% glutaraldehyde solution (final concentration) was used to fix the membrane before air-drying at ambient temperature. The control PE sheets that were cultured in sterile Gause’s medium were also treated using the same procedure. Consequently, the surface texture of all PE sheets was characterized by scanning electron microscope (SEM) (Tescan Vega 3 LMH, Tescan Corporation, Brno, Czech Republic). Elongation to fracture and tensile strengths were measured at a rate of 50 mm/min by a tensile tester ((Instron 3367, Instron Corporation, Norwood, MA, USA)), and the results were recorded as average of three tests.

### 2.3. Biodegradation of Polyethylene (PE)

PE powder samples (Sinopec Maoming petrochemical company, Maoming, Guangdong, China) with a molecular weight (MW) of 5000 Da and 10,000 Da were treated by 75% ethanol for 2 h and dried under sterile conditions, then sterilized by ultraviolet radiation for 2 h, during which the samples were stirred every half hour to sterilize them thoroughly. The sterilized PE powder was added to the sterile water and the washed water was plated onto the plates to determine whether it was completely sterilized. Two kinds of PE powder specimens were separately added to the sterilized 50 mL carbon-free Gause’s medium. 0.5 mL bacterial cultures (*S. albogriseolus* LBX-2 and *E. coli* BL21(DE3)) with the absorbance value at OD600 of 0.6 were inoculated into the above Gause’s medium, respectively. The cultures were prepared for 15 days at 37 °C, 180 rpm. All the experiments were performed in triplicate. The cultures were filtered, and then the filtered mixtures were added to a 2% SDS solution and ultrasonicated by ultrasonic cleaner (Kunshan Shumei Co., Ltd., Kunshan, Jiangsu, China) for 2 h. Subsequently, the samples were placed at 37 °C for 12 h and the PE powder floating on the liquid surface was separated, dried, and weighed.

### 2.4. Growth in Octadecane and Hexadecane

The liquid n-hexadecane and n-octadecane (Aladdin, Shanghai, China) were filtered through a 0.22 μm membrane into 50 mL carbon-free Gause’s medium at a final concentration of 1% and 3%, respectively. 0.5 mL bacterial cultures (*S. albogriseolus* LBX-2 and *E. coli* BL21(DE3)) with the absorbance value of 0.6 at OD600 were inoculated into the medium. The cultures were incubated at 37 °C, 180 rpm, for 7 days. At the indicated time points, the cultures were sampled for the determination of the cell density.

### 2.5. Genomic DNA Extraction

A colony of *S. albogriseolus* LBX-2, which has been isolated by our lab, was transferred to new Gause’s agar plates. Then, 1 mL of the culture grown overnight was further inoculated in 100 mL liquid medium and incubated at 37 °C, with shaking at 180 rpm until its OD600 value was about 0.6. The cells were collected by centrifugation at 8000 rpm, and the cells were removed. Pelleted cells were mixed with quartz sand and thoroughly ground in liquid nitrogen. The total DNA was extracted with a TIANamp Bacteria DNA Kit (Tiangen Biotech Co., Ltd., Beijing, China) according to the manufacturer’s suggestions. The size of the genomic DNA was estimated by agarose. The purity and concentration of the genomic DNA were then assessed with a spectrophotometer Nanodrop 2000 (Wilmington, DE, USA). A total of 300 ng of the genomic DNA was used to prepare for the DNA Library (PacBio, PacBio RSII, Menlo Park, CA, USA and Illumina Inc., Illumina Hiseq, San Diego, CA, USA).

### 2.6. Genome Sequencing and Annotation

The genome of *S. albogriseolus* LBX-2 was sequenced at the Beijing Novogene Bioinformatics Technology Co., Ltd. using a 10 kb SMRTbell™ template library using the Pacific Biosciences (PacBio) RSII Single Molecule Real Time (SMRT) sequencing platform. The low-quality reads were routinely cut using the SMRT 2.3.0 and the remaining reads were assembled to generate one contig without gaps. The predicted complete coding sequences (CDSs) were translated and used to search against the NCBI non-redundant (NR), Clusters of Orthologous Groups (COG), Swiss-Prot, TrEMBL, Transporter Classification Database (TCDB), Kyoto Encyclopedia of Genes and Genomes (KEGG) and Gene Ontology (GO) databases using the BLASTP restricted parameters (i.e., E-value less than 1e-5, minimal2 alignment length of > 40%). Clustered, regularly interspaced, short palindromic repeats were identified by CRISPRF inder. Noncoding RNA, such as transfer RNA (tRNA), ribosome RNA (rRNA) and small nuclear RNAs (snRNA) were predicted by tRNAscan-SE, rRNAmmer and Rfam, respectively. The secretory proteins were identified by the SignalP 4.1, and the prediction of Type I–VII proteins secreted by the pathogenic bacteria was based on the EffectiveT3. The Pfam database was used for protein domain analysis and the TMpred was utilized to predict the transmembrane helices of oxygenase.

### 2.7. Phylogenetic Analysis

The 16S rRNA gene used to identify the species was obtained by BLASTn search against the whole genome of *S. albogriseolus* LBX-2. Phylogenetic analysis of the 16S rRNA gene sequences was generated by MEGA (MEGA6.0, Tokyo Metropolitan University, Tokyo, Japan) using the Neighbor-Joining method with 1,000 bootstraps for orthologous genes. The other 16S rRNA sequences used in the phylogenetic tree were downloaded from the NCBI database, including *S. albogriseolus* NRRL B-1305 (AJ494865.1), *S. albogriseolus* ABRIINW EA1145 (GQ925802.1), *Actinobacterium* HSr13 (KP247554.1), *Streptomyces viridodiastaticus* NBRC 13106 (NR_112371.1), *Actinomycetales bacterium* XJSS-21 (EU598255.1), *Actinobacterium* HSr11 (KP247553.1), *Trichotomospora caesia* (AB006154.1), *Kitasatospora paracochleata* (AB022873.1), *Kitasatospora papulosa* JCM 7250 (NR_126320.1), *Kitasatospora paracochleata* NBRC 14769 (NR_112443.1), *Streptomyces aureus* IN144 (KT923349.1).

## 3. Results

### 3.1. Incubation Description

**Figure 1:**
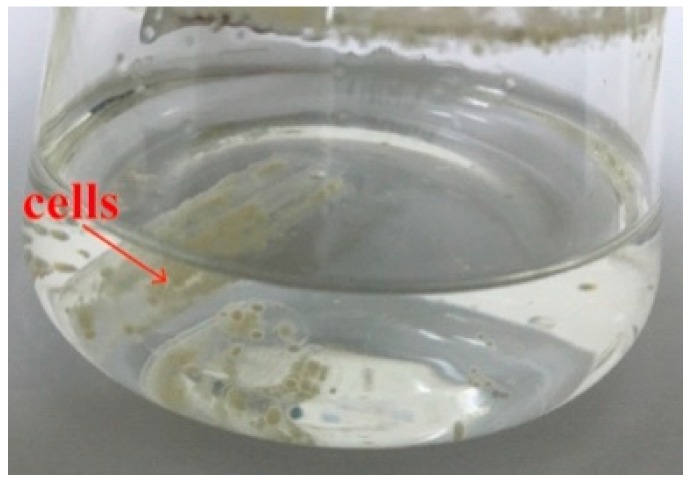
*S. albogriseolus* LBX-2 in basal medium with polyethylene (PE) membrane as the sole carbon source. The yellow cells indicated by the red arrow are *S. albogriseolus* LBX-2.

The *S. albogriseolus* LBX-2 was inoculated into the medium with PE membrane serving as the sole carbon source. Results showed that the liquid appeared to be turbid after 7 days, while the PE film curled with many cells was observed to attach on the plastic’s surface after 15 days (Figure 1).

### 3.2. Micro-Scale Analysis of the Surface

**Figure 2:**
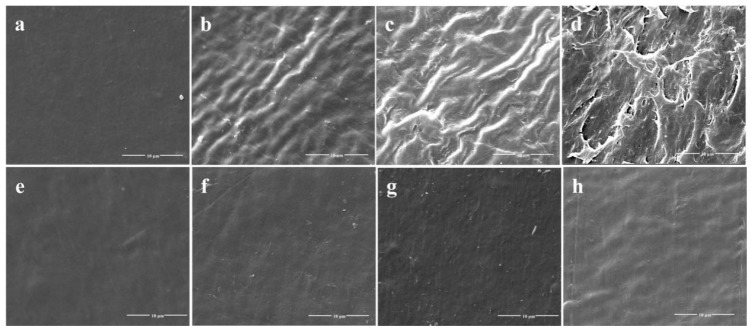
Scanning electron microscopy (SEM) observations of the physical surface topography of the untreated PE sheets versus PE sheets treated with strain LBX-2 after 0, 15, 30, 45 days, respectively. (**a**–**d**) Physical changes on the PE film sheets incubated with strain LBX-2 after 0, 15, 30, 45 days, respectively. (**e**–**h**) Physical changes on the PE film sheets without strain LBX-2 after 0, 15, 30, 45 days, respectively. Scale bar = 10 μm.

After a 45-day incubation, the surface change of all PE films was observed by SEM. PE membranes immersed in cultures without *S. albogriseolus* LBX-2 were used as the control. It was found that the surface of the treated PE films showed wrinkles and dents at day 15, with significant observable damages at day 30 (Figure 2). Pits and cavities were observed after 45 days, which could be associated with bacterial activity as compared with the control. The SEM observations suggest that the *S. albogriseolus* LBX-2 could have caused the changes and damages to the PE’s physical structure.

### 3.3. Tensile Performance

**Figure 3:**
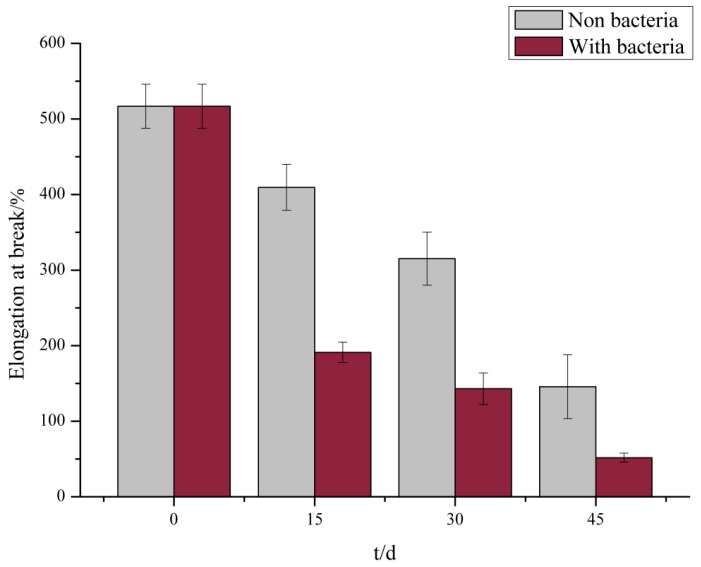
Measured results of elongation at break of membrane control group and experimental group.

The tensile test demonstrated that no significant change occurred in the elongation at the break of the PE films at the initial stage but was reduced by 63% after incubation with *S. albogriseolus* LBX-2 for only 15 days. In comparison, those in the control were only reduced by 20.8% (Figure 3). Similarly, the elongation at breaking point of the treated membrane after 45 days was reduced by 90%, which was significantly higher than that of the control. These results indicate that albeit the long-term immersion in the medium itself reduced the elongation at break of PE, but the treatment with *S. albogriseolus* LBX-2 is able to dramatically accelerate the decomposition of polyethylene.

### 3.4. Biodegradation Assays

**Figure 4:**
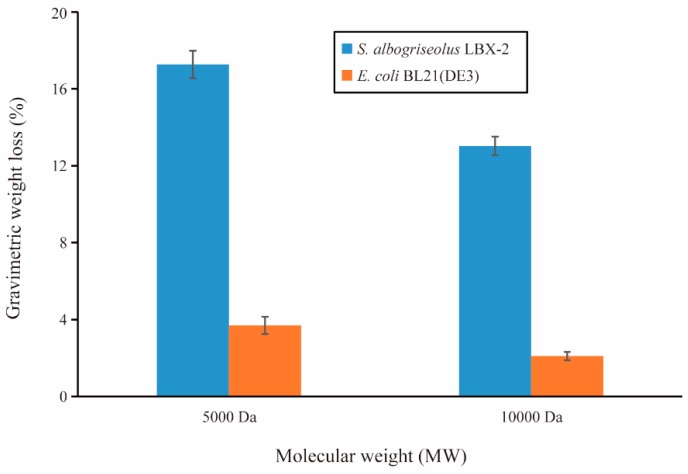
PE biodegradation by strains LBX-2 and *E. coli* BL21(DE3). Changes in the gravimetric weight of the PE powder over the 15-day period (mean value ± standard deviation (SD), *n* = 3).

The weight loss over time of the PE powder inoculated with the strain LBX-2 and *E. coli* BL21(DE3) is presented in Figure 4. During a 15-day incubation period in Gause’s medium containing PE powder as the sole carbon source, the incubation with strain LBX-2 resulted in a net loss in the PE samples of 17.27 ± 0.48 and 13.03 ± 0.74 for 5000 Da and 10,000 Da PE powders, respectively. While when treated with *E. coli* it was only 3.7 ± 0.22 and 2.1 ± 0.31. It suggests that the weight loss rate of PE treated with LBX-2 is significantly more than that of the control.

### 3.5. Utilization of Alkane by S. Albogriseolus LBX-2

**Figure 5:**
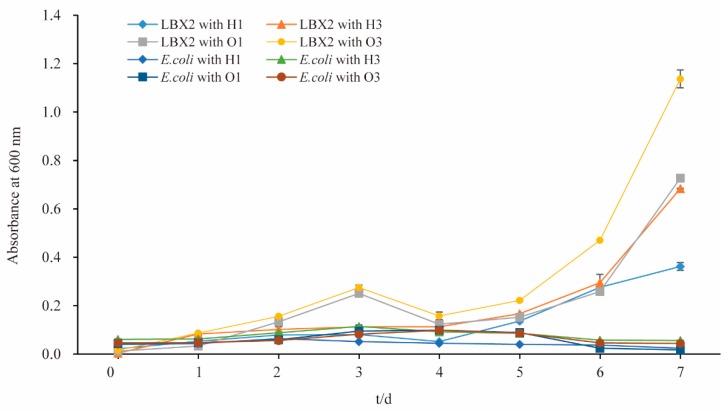
Growth curves of *S. albogriseolus* LBX-2 and *E. coli* BL21(DE3) in the medium containing n-hexadecane or n-octadecane. H1: the final concentration of n-hexadecane was 1%, H3: the final concentration of n-hexadecane was 3%, O1: the final concentration of n-octadecane was 1%, O3: the final concentration of n-octadecane was 1%.

Growth curve results showed that the strain LBX-2 could grow in the medium containing hexadecane or octadecane as the sole carbon source in Figure 5. This strain had a better growth in n-octadecane than n-hexadecane, and better growth in high concentration (3%) than low concentration (1%). However, the negative control strain *E. coli* BL21(DE3) could not grow in both n-hexadecane and n-octadecane. This indicates that the LBX-2 strain can utilize not only PE but also some other alkanes as the carbon source.

### 3.6. Whole Genome Characterization

**Figure 6:**
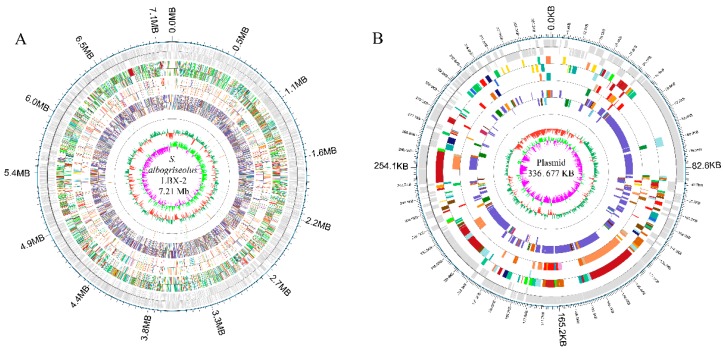
Genomic architecture of *S. albogriseolus* LBX-2. (**A**) Genome map of *S. albogriseolus* LBX-2 and (**B**) Plasmid map of *S. albogriseolus* LBX-2. Each ring, from outside to the center represented the following features: annotation of functional gene, Clusters of Orthologous Groups (COG) categories, Kyoto Encyclopedia of Genes and Genomes (KEGG) categories, Gene Ontology (GO) categories, ncRNA, GC content, and guanine and cytosine (GC) content skew.

*S. albogriseolus* LBX-2 has a linear chromosome which is 7,210,477 bp in size with a GC content of 72.47% (bioproject accession: PRJNA558864) (Figure 6). Also, it harbors a linear plasmid with a size of 336,677 bp and a GC content of 73.66%. Analysis of 333 *Streptomyces* strains in the NCBI database revealed that the N50 value of *Streptomyces* genome was 8.27 Mb with an average of 8.41 Mb, and an average GC content of 71.54% (Table 1). In comparison, the genome size of *S. albogriseolus* LBX-2 was lower than the average size for *Streptomyces* but had a higher GC content (72.47%). The number of proteins encoded by these 311 *Streptomyces* strains was also counted. *Streptomyces regensis* (ID: 38737) encodes the largest number of proteins (12,910 predicted proteins), while *Streptomyces regensis* (ID: 38737) encodes the least (4043 proteins). Thirty-four strains harbors 1 to 7 plasmids, and *Streptomyces autolyticus* (ID: 52894) contained the most numbers of plasmid up to 7. It was revealed that *S. albogriseolus* LBX-2 has only one plasmid. A total of 6704 CDS were predicted as well as 86 ncRNAs, 67 tRNAs, 18 rRNAs and 1 sRNA, and 4474, 4918, 3064, 2157 and 6531 genes were annotated by GO, COG, KEGG and NR databases, respectively. In addition, a total of 38 repeats, 22 genomics islands, 9 pre-phages, and 3 CRISPR sequences were identified in *S. albogriseolus* LBX-2 (Table 2).

### 3.7. DNA Methylation

DNA methylation is an important epigenetic modification process that plays a key role in gene expression, cell cycle, anti-mutation and stability of genome [22,23]. In this study, the appreciation modification module of the SMRT Portal software was used to predict methylation sites and motifs for the final genome assembly. Here, we observed that *S. albogriseolus* LBX-2 had 2,541,603 predicted DNA methylation sites, of which the unknown type accounted for 79.35%, followed by m4C (20.36%) and m6A (0.29%).

### 3.8. Oxygenase Genes of S. Albogriseolus LBX-2

Oxygenases have been suggested to be essential enzymes involved in the degradation of PE or other alkane, although its degradation mechanisms remain unclear [2]. The oxygenase gene sequences (including both monooxygenase and dioxygenase) were analyzed by sequence alignment and annotation. It was observed that there were 53 oxygenase genes in the genome of *S. albogriseolus*, of which 21 were monooxygenases and 21 were dioxygenases (Supplementary Table S1). Signal peptide prediction was performed by Signal IP 4.1 and revealed that none of these 53 oxygenases had a signal peptide. Based on the Pfam database, 36 oxygenases were predicted to contain one or several transmembrane helices and 18 of the 21 monooxygenases have transmembrane helices.

### 3.9. Phylogenetic Analysis

**Figure 7:**
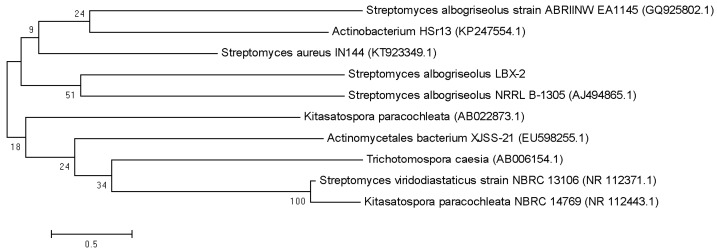
Phylogenetic tree showing the position of *S. albogriseolus* LBX-2.

The nearly full-length sequence (1517 bp) of the 16S rRNA gene (NCBI accession number: MN338058) of *S. albogriseolus* LBX-2 was extracted from the genome by BLAST. The results showed that *S. albogriseolus* LBX-2 clustered with the other Streptomyces species in one clade. LBX-2 was most closely related to S. albogriseolus NRRL B-1305 with shared 100% homology (Figure 7).

### 3.10. Synteny Comparison with the Genome of Streptomyces Coelicolor A3(2)

**Figure 8:**
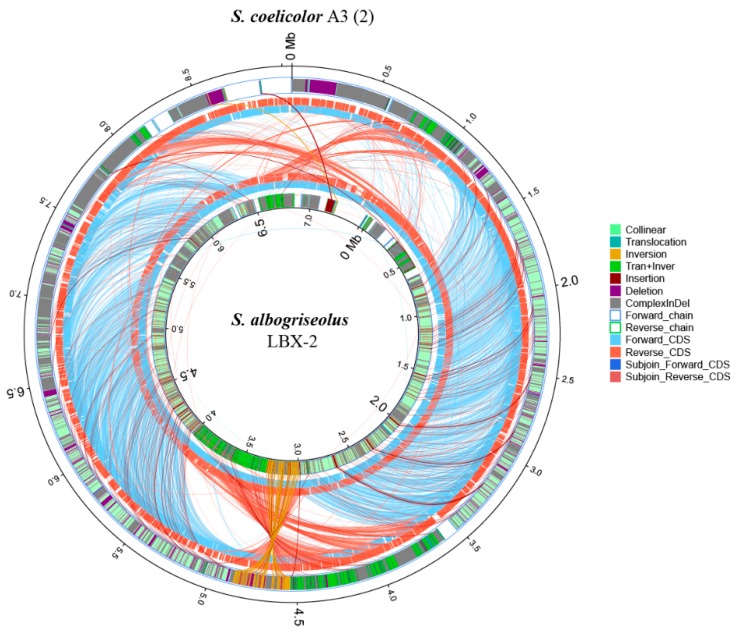
Synteny comparison of *S. albogriseolus* LBX-2 genome with the genome of the type strain *S. coelicolor* A3(2). Outer circle: *S. coelicolor* A3(2) genome; Inner circle: *S. albogriseolus* LBX-2 genome; Collinear: collinear region; Translocation: translocation region; Inversion: Iinversion region; Tran + Inver: translocation and inversion region; Insertion: insertion region; Deletion: deleted region (≥ 50 bp); Complex Indel: the region that corresponds to a position but cannot be compared; Forward chain: the forward chain of the genome sequence, the gene coordinates increased clockwise; Reverse chain: the reverse chain of the genome sequence, the gene coordinates increased counterclockwise; Forward CDS: CDS translated from the forward chain; Reverse CDS: CDS translated from the reverse chain; Subjoin forward CDS: the subjoin of CDS translated from the forward chain; Subjoin Reverse CDS: the subjoin of CDS translated from the reverse chain (Related notes information cited in Reference [26]).

*S. coelicolor* A3(2) is the best genetically characterized *Streptomycete* which was a frequently used as a model organism for morphological, antibiotic production and physiological differentiation studies [24,25]. Phylogenetic analysis of *Streptomyces* strains revealed that the strain *S. albogriseolus* LBX-2 was also closely related to *S. coelicolor* A3(2) (98.62% 16s RNA gene identity), with 60.12% of their genomes in synteny (Figure 8). However, there were many complex-indel, deletion, trans + inver, insertion, translocation and inversion between *S. albogriseolus* LBX-2 and *S. coelicolor* A3(2). At the same time, SNP sequence analysis showed that *S. albogriseolus* LBX-2 had 436,472 SNP loci was higher when compared with the reference genome, which only had 245,040 synonymous sites and 163,223 non-synonymous sites, which were all found in the CDS region.

## 4. Discussion

The most produced and consumed artificial polymer, PE, has already generated a massive volume of improperly managed wastes that are directly dumped into the environment causing threats and damages to both terrestrial and marine wildlife [27]. Many studies reported the potential of microorganisms in degrading PE. However, only a few have been isolated using PE as the sole carbon source [28]. Previous studies have demonstrated that the degradation of PE by most microorganisms relied on pretreatment (photo- or thermal-based pretreatments) for better degradation [29]. However, it is difficult to carry out such physical pretreatments on the plastic waste under natural conditions. Therefore, it is important to find the microorganisms that could directly degrade the virgin PE film as a carbon source even without UV or high-temperature pre-degradation.

In this study, we successfully analyzed and annotated the complete genome profiles of a PE degrading strain *S. albogriseolus* LBX-2. Similar to the strain obtained by Yang Jun et al. [29], *S. albogriseolus* LBX-2 can directly use the untreated PE films as the sole carbon source, causing pits and cavities in the surface of the plastics suggesting enzyme-assisted degradation. The mechanical properties of PE membrane were also dramatically altered after 45 days of co-incubation with the bacteria. During a 15-day incubation, the weight loss of the PE specimens with the strain LBX-2 inoculation increased significantly. In addition, LBX-2 is more preferable to degradation of 5000 Da PE powder than that of 10,000 Da. However, a 15-day PE degradation was performed due to abundant LBX-2 cells which could adhere to the surface of the PE powder, which was difficult to remove in longer incubation. Therefore, only a 15-day PE degradation was performed. In fact, using the weight loss rate of plastic powder or membrane to assess the degradation of polyethylene is not an ideal method. It is better to measure the conversion of the plastic’s carbon into CO_2_ (or CO_2_ and CH_4_), while the specific incubation conditions (e.g., time, temperature, relative humidity) and the key polymer-specific properties should also be considered [30,31].

The genome sequence of *S. albogriseolus* LBX-2 contains a linear chromosome of 7,210,477 bp with a GC content of 72.47%. There was one plasmid that had 336,677 bp in the genome, suggesting that it could be altered to establish a plasmid-based expression system. Compared with the other members of this bacterial genus, the genome size of *S. albogriseolus* LBX-2 was lower than the average but its GC content (72.47%) was higher.

The specific mechanism underlying polyethylene microbial biodegradation remains unclear, although many studies demonstrated the capacity of bacteria for the biodegradation of alkane, which has a similar chemical structure to polyethylene [32]. Further findings indicate that some enzymes in these microorganisms may play a major role in the biodegradation of alkane [33]. The alkane hydroxylase system was also investigated in their role in degrading alkanes via alkane oxygenase, which participates in the first step in the pathway and plays in the hydroxylation of the terminal carbon of alkanes [27,34,35]. Alkane monooxygenase that catalyzes alkane biodegradation with the involvement of electron transport proteins has been recognized as one of the key enzymes in PE degradation. The monooxygenase genes that may have contributed to the degradation of alkane by this bacterium were also identified. Unlike other bacterial strains which only have a few alkane monooxygenase genes such as *Pseudomonas putida* GPo1 that had only one [34], *Rhodococcus* sp. For TMP2 having 5 alkane monooxygenase genes (AlkB1, AlkB2, AlkB3, AlkB4 and AlkB5) [36], 21 monooxygenase genes were found in the genome of *S. albogriseolus* LBX-2. This indicates the strong degradation ability of PE by *S. albogriseolus* LBX-2. Besides, growth curve tests in this research show that LBX-2 actually have the ability to degrade some other alkanes.

We also analyzed the signal peptide sequence of these oxygenase and found that all monooxygenases and dioxygenases have no signal peptides. However, most of the monooxygenases contain the predicted transmembrane helices, indicating that they may mainly act transmembranely while participating in plastic degradation, but the specific mechanism remains unclear. Furthermore, based on the known functions of the two alkane monooxygenase genes in *Alcanivorax borkumensis* SK2, it can be seen that different alkane monooxygenase genes may play different functions and could also be induced by different factors for gene expression [37]. Therefore, functional verification of these genes is needed to understand their specific roles in plastics degradation. Based on these observations, the ARTP mutagenesis, genome resequencing, and RNA-sequencing techniques could be utilized to further study *S. albogriseolus* LBX-2 and develop its potential use in the environmental industry.

## Figures and Tables

**Table 1:** Genomic information of *Streptomyces*.

Features	Maximum	Minimum	Average	N50
Genome size	15.09 Mb	2.09 Mb	8.41 Mb	8.27 Mb
GC content	74.8%	67.2%	71.54%	71.6%
Protein number	12,910	4043	7031	6892
Plasmid number	7	1	1.88	2

**Table 2:** General features associated with the genome in *S. albogriseolus* LBX-2.

Attribute	Value	% of Total (%)
Genome size	7,547,154	100%
DNA coding number of bases	6,597,501	87.42%
DNA G + C number of bases	5,501,120	72.89%
Genes	6704	100.00%
Genes of nuclear DNA	6477	96.61%
Genes of plasmid	227	3.39%
Total length of genes	6,597,501	87.42%
Repeat	38	100.00%
Long terminal repeat (LTR)	19	50.00%
DNA repeat elements (DNA)	6	15.79%
Long interspersed nuclear elements (LINE)	6	15.79%
Short interspersed nuclear elements (SINE)	5	13.16%
Rolling circle (RC)	2	5.26%
CRISPR count	3	100.00%
CRISPR of nuclear DNA	2	66.67%
CRISPR of plasmid	1	33.33%
Genomics islands	22	100.00%
Genomics Islands of nuclear DNA	21	95.45%
Genomics Islands of plasmid	1	4.55%
pre phage	9	100.00%
pre phage of nuclear DNA	8	88.89%
pre phage of plasmid	1	11.11%
Non-coding RNA (ncRNA)	86	100.00%
Transfer RNA (tRNA)	67	77.91%
5S	6	6.98%
16S	6	6.98%
23S	6	6.98%
Small RNA (sRNA)	1	11.63%
Gene annotation	--	--
Genes assigned to GO	4474	66.74%
Genes assigned to COG	4918	73.36%
Genes assigned to KEGG	3064	45.70%
Genes assigned to SwissProt	2157	32.17%
Genes assigned to NR	6531	97.42%

## Supplementary Materials

The following are available online at https://www.mdpi.com/2076-2607/7/10/379/s1. The complete genome and plasmid sequence can be retrieved using the BioProject number: PRJNA558864. The 16S rRNA sequence can be obtained by the accession number: MN338058. Table S1: The oxygenase proteins in the *S. albogriseolus* LBX-2.
