# Supplementary material for: Complete Genome Sequence and Characterization of a Polyethylene Biodegradation Strain, Streptomyces Albogriseolus LBX-2
Source: Microorganisms. 2019 Sep 22;7(10):379. doi: 10.3390/microorganisms7100379 (PMC6843780; doi:10.3390/microorganisms7100379)
Supplement: Supplementary file 1 [file microorganisms-07-00379-s001.pdf]

Table S1. The oxygenase proteins in the *S. albogriseolus* LBX-2

Gene_id	Accession number in NCBI	Subject description	Length of CDS	Protein length(aa)	Domain	Predicted transmembrane helices
SA_2GM000034	WP_030835026.1	cysteine dioxygenase	462	153	CDO_I	0
SA_2GM000124	KEG44335.1	antibiotic biosynthesis monooxygenase	333	110	ABM	0
SA_2GM000173	KEG44118.1	FAD-binding monooxygenase	1185	394	NAD_binding_8<br>FAD_binding_3	2
SA_2GM000470	KEG43823.1	extradiol dioxygenase	393	130	Glyoxalase	1
SA_2GM000547	WP_030862881.1	monooxygenase	1821	606	FAD_binding_3	2
SA_2GM000595	WP_006143212.1	aromatic ring-opening dioxygenase LigA	768	255	N/A	1
SA_2GM000825	WP_031016361.1	4-hydroxybenzoate 3-monooxygenase	1164	387	FAD_binding_3	1
SA_2GM000846	WP_031016338.1	monooxygenase	1281	426	FAD_binding_3	2
SA_2GM001217	KEG43086.1	homogentisate 1, 2-dioxygenase	1362	453	HgmA	0
SA_2GM001483	WP_019525293.1	dioxygenase	429	142	Glyoxalase	0
SA_2GM001560	WP_006136098.1	glyoxalase/bleomycin resistance protein/dioxygenase	399	132	Glyoxalase	0
SA_2GM001769	WP_028959795.1	heme oxygenase	648	215	Heme_oxygenase	0
SA_2GM002219	WP_030934652.1	dimethylaniline monooxygenase	1266	421	Pyr_redox_3	4
SA_2GM002319	WP_019525156.1	FMN-linked alkanal monooxygenase	1095	364	Bac_luciferase	2
SA_2GM002409	WP_026243851.1	4-hydroxyphenylpyruvate dioxygenase	1146	381	Glyoxalase	0
SA_2GM002516	WP_019527097.1	cysteine dioxygenase	534	177	CDO_I	0
SA_2GM002566	WP_019522668.1	4-hydroxybenzoate 3-monooxygenase	1176	391	FAD_binding_3	1
SA_2GM002661	WP_006135839.1	monooxygenase	1518	505	FMO-like	3
SA_2GM002695	KEG41654.1	extradiol ring-cleavage dioxygenase	786	261	LigB	1
SA_2GM002739	KEG41618.1	monooxygenase	1200	399	Pyr_redox_3	1

SA_2GM003219	WP_019526616.1	phenylacetate-CoA oxygenase subunit PaaI	738	245	PaaA_PaaC	0
SA_2GM003220	WP_024886305.1	phenylacetate-CoA oxygenase	288	95	PaaB	0
SA_2GM003221	WP_019526614.1	phenylacetate-CoA oxygenase subunit PaaA	1017	338	PaaA_PaaC	1
SA_2GM003294	KEG41105.1	antibiotic biosynthesis monooxygenase	321	106	ABM	0
SA_2GM003390	WP_028959522.1	tryptophan 2, 3-dioxygenase	831	276	Trp_dioxygenase	0
SA_2GM003892	WP_015655068.1	glyoxalase/bleomycin resistance protein/dioxygenase	369	122	Glyoxalase	0
SA_2GM004018	WP_006133448.1	FAD-binding monooxygenase	1224	407	FAD_binding_3	2
SA_2GM004037	KEG40385.1	pentachlorophenol monooxygenase	1401	466	FAD_binding_3	1
SA_2GM004397	KEG40007.1	oxygenase	1263	420	Smoa_sbd	1
SA_2GM004480	WP_026243711.1	alkane 1-monooxygenase	1008	335	Bac_luciferase	1
SA_2GM004850	KEG39638.1	monooxygenase	1620	539	FAD_binding_3	2
SA_2GM004918	KEG39353.1	monooxygenase	2094	697	FMO-like adh_short	2
SA_2GM004921	KEG39356.1	glyoxalase/bleomycin resistance protein/dioxygenase	804	267	Glyoxalase_6	1
SA_2GM004947	WP_030835026.1	cysteine dioxygenase	462	153	CDO_I CDO_I	0
SA_2GM005813	WP_024883684.1	phytanoyl-CoA dioxygenase	1158	385	PHYH	1
SA_2GM005885	WP_028959095.1	monooxygenase	1614	537	FAD_binding_3	2
SA_2GM005890	WP_024883620.1	3-(2, 3-dihydroxyphenyl)propionate dioxygenase	936	311	LigB	3

SA_2GM005903	WP_009302965.1	putative nitric oxide dioxygenase	1215	404	Globin<br>FAD_binding_6<br>NAD_binding_1	1
SA_2GM005948	WP_024883567.1	glyoxalase/bleomycin resistance protein/dioxygenase	372	123	Glyoxalase	1
SA_2GM006054	KEG38411.1	extradiol dioxygenase	393	130	Glyoxalase_6	0
SA_2GM006067	KEG38425.1	lactate 2-monooxygenase	1170	389	FMN_dh	1
SA_2GM006372	KEG44444.1	phenylacetate-CoA oxygenase	1032	343	PaaA_PaaC	1
SA_2GM006376	KEG44440.1	phenylacetate-CoA oxygenase	1140	379	FAD_binding_6<br>NAD_binding_1<br>Fer2	1
SA_2GM006378	KEG44438.1	tryptophan 2, 3-dioxygenase	849	282	Trp_dioxygenase	1
SA_2GM006397	WP_028803713.1	alkane 1-monooxygenase	1086	361	FA_desaturase	5
SA_2GM006509	WP_005321566.1	cytochrome P450 monooxygenase	1230	409	p450	1
SA_2GM006535	CBW45725.1	putative oxygenase	1356	451	FAD_binding_3	1
SA_2GM006559	WP_005321822.1	putative oxygenase	309	102	ABM	0
SA_2GM006560	CBW45655.1	putative oxygenase	1761	586	FAD_binding_3	2
SA_2GM006567	BAK86399.1	phenylacetyl-CoA dioxygenase	1404	467	ECH_1	1
SA_2GM006611	WP_009945666.1	2-nitropropane dioxygenase, NPD	1581	526	PfaD_N<br>NMO	2
SA_2GM006676	KFG72918.1	monooxygenase	324	107	ABM	0
